# The current distribution of tick species in Inner Mongolia and inferring potential suitability areas for dominant tick species based on the MaxEnt model

**DOI:** 10.1186/s13071-023-05870-6

**Published:** 2023-08-16

**Authors:** Rui Ma, Chunfu Li, Haoqiang Tian, Yan Zhang, Xinyu Feng, Jian Li, Wei Hu

**Affiliations:** 1 College of Life Sciences, Inner Mongolia University, Hohhot, 010070 China; 2 National Institute of Parasitic Diseases, Chinese Center for Disease Control and Prevention, Key Laboratory of Parasite and Vector Biology of China Ministry of Health, WHO Collaborating Centre for Tropical Diseases, Joint Research Laboratory of Genetics and Ecology on Parasite-Host Interaction, Chinese Center for Disease Control and Prevention, Fudan University, Shanghai, 200025 China; 3 School of Global Health, Chinese Center for Tropical Diseases Research, Shanghai Jiao Tong University School of Medicine, Shanghai, 20025 China; 4 One Health Center, Shanghai Jiao Tong University-The University of Edinburgh, Shanghai, 20025 China; 5 Basic Medical College, Guangxi Traditional Chinese Medical University, Nanning, 530005 Guangxi China; 6 Department of Infectious Diseases, Huashan Hospital, State Key Laboratory of Genetic Engineering, Ministry of Education Key Laboratory for Biodiversity Science and Ecological Engineering, Ministry of Education Key Laboratory of Contemporary Anthropology, School of Life Sciences, Fudan University, Shanghai, 200438 China

**Keywords:** Tick, MaxEnt model, Suitability area, Environmental factor, Inner Mongolia

## Abstract

**Background:**

Ticks are known to transmit a wide range of diseases, including those caused by bacteria, viruses, and protozoa. The expansion of tick habitats has been intensified in recent years due to various factors such as global warming, alterations in microclimate, and human activities. Consequently, the probability of human exposure to diseases transmitted by ticks has increased, leading to a higher degree of risk associated with such diseases.

**Methods:**

In this study, we conducted a comprehensive review of domestic and international literature databases to determine the current distribution of tick species in Inner Mongolia. Next, we employed the MaxEnt model to analyze vital climatic and environmental factors influencing dominant tick distribution. Subsequently, we predicted the potential suitability areas of these dominant tick species under the near current conditions and the BCC-CSM2.MR model SSP245 scenario for the future periods of 2021–2040, 2041–2060, 2061–2080, and 2081–2100.

**Results:**

Our study revealed the presence of 23 tick species from six genera in Inner Mongolia, including four dominant tick species (*Dermacentor nuttalli*, *Ixodes persulcatus*, *Dermacentor silvarum*, and *Hyalomma asiaticum*). *Dermacentor nuttalli*, *D. silvarum*, and *I. persulcatus* are predominantly found in regions such as Xilin Gol and Hulunbuir. Temperature seasonality (Bio4), elevation (elev), and precipitation seasonality (Bio15) were the primary variables impacting the distribution of three tick species. In contrast, *H. asiaticum* is mainly distributed in Alxa and Bayannur and demonstrates heightened sensitivity to precipitation and other climatic factors. Our modeling results suggested that the potential suitability areas of these tick species would experience fluctuations over the four future periods (2021–2040, 2041–2060, 2061–2080, and 2081–2100). Specifically, by 2081–2100, the centroid of suitable habitat for *D. nuttalli*, *H. asiaticum*, and *I. persulcatus* was predicted to shift westward, with new suitability areas emerging in regions such as Chifeng and Xilin Gol. The centroid of suitable habitat for *H. asiaticum* will move northeastward, and new suitability areas are likely to appear in areas such as Ordos and Bayannur.

**Conclusions:**

This study provided a comprehensive overview of the tick species distribution patterns in Inner Mongolia. Our research has revealed a significant diversity of tick species in the region, exhibiting a wide distribution but with notable regional disparities. Our modeling results suggested that the dominant tick species’ suitable habitats will significantly expand in the future compared to their existing distribution under the near current conditions. Temperature and precipitation are the primary variables influencing these shifts in distribution. These findings can provide a valuable reference for future research on tick distribution and the surveillance of tick-borne diseases in the region.

**Graphical Abstract:**

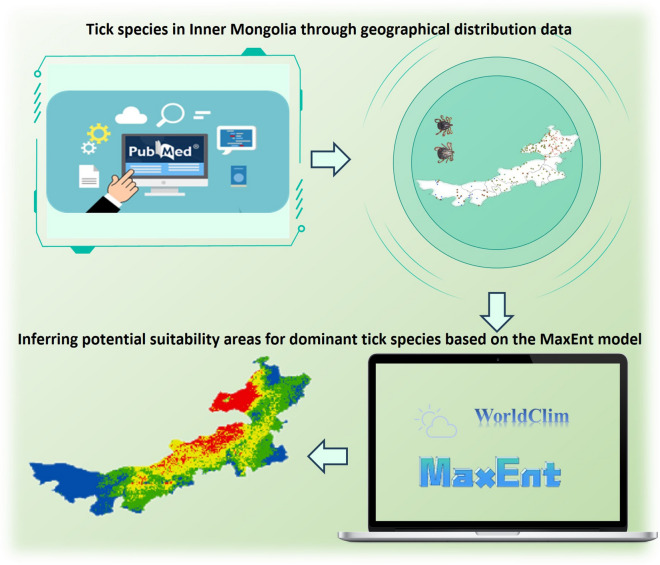

**Supplementary Information:**

The online version contains supplementary material available at 10.1186/s13071-023-05870-6.

## Background

Ticks are hematophagous arthropods that parasitize various vertebrate hosts, including livestock and wildlife [1]. Approximately 900 species of ticks worldwide are classified into three families and 18 genera. The reported presence of 125 tick species in China, consisting of 111 hard ticks (Ixodidae) and 14 soft ticks (Argasidae), serves as compelling evidence of the striking heterogeneity of tick diversity within the country [2]. Ticks not only are significant ectoparasites of animals but also serve as vectors and reservoirs for a wide range of important pathogens affecting humans and animals [3]. Ticks require blood meals to complete their life cycle, but this process can also cause localized infection, edema, acute inflammation, and secondary infections in the host. Additionally, ticks can transmit various tick-borne pathogens, including bacteria, viruses, and protozoans, which pose a significant threat to human and animal health [4, 5]. With global climate change and the expansion of human activities, tick bite incidents and associated infections have become a significant public health threat with increasingly severe consequences.

Due to the unique physiological habits of ticks, they spend most of their life cycle in natural environments outside their hosts. Consequently, ecological conditions are closely related to tick distribution patterns and population abundance. Conversely, climatic factors and host diversity can be used to predict areas at risk for tick-borne diseases [6]. Increased temperatures can positively impact ticks’ subsistence and reproduction during their non-parasitic life stages, promoting population growth and the possibility of expansion, establishment, and survival in new geographical areas [7]. Global climate change also indirectly affects tick-borne disease transmission, such as influencing vegetation distribution, the abundance of pathogen reservoir hosts, human behavior, and land use, which affect tick abundance and pathogen transmission [8]. Therefore, under the increasing risk of tick-borne diseases with climate change, understanding the distribution of dominant tick species under current and future climate conditions is profoundly significant for the prevention and control of tick-borne disease [9].

Several models have been developed to predict the distribution of species, including the maximum entropy (MaxEnt) model, classification and regression tree (CART) model, generalized linear model (GLM), habitat model (HABITAT), genetic algorithm for rule-set prediction (GARP) model, and bioclimatic model (BIOCLIM) [10, 11]. Among these, the MaxEnt model is based on the theory of species ecological niches and uses the location information of species occurrence and environmental background data to fit the probability distribution with the MaxEnt value, estimating the potential species distribution. Under the premise of known partial information, the most reasonable inference about the unknown distribution is the least specific or most random inference that conforms to the known information. The MaxEnt model has several advantages, such as using existing incomplete, small-sample, and discrete distribution data to predict species’ suitable distribution with high accuracy, stability, and easy interpretation [12, 13]. Therefore, it is widely used to predict ticks’ suitable habitat distribution.

Inner Mongolia, the widest province in China by latitude, is located along the northern border and is currently a high-incidence area for tick-borne diseases. Various tick-borne diseases, such as rickettsiosis, Crimean-Congo hemorrhagic fever, and brucellosis, have been reported in the region [14–16]. Inner Mongolia's unique natural climate, nomadic lifestyle, and ecotones between forests and grasslands provide favorable conditions for tick subsistence and reproduction. The rapidly developing livestock industry has significantly altered the interactions between animals and their habitats, making the relationship between ticks and host animals more complex. Moreover, multiple border ports in Inner Mongolia increase the risk of tick expansion and cross-border transmission of pathogens due to human activities and international trade. This study predicted the distribution of dominant tick species in Inner Mongolia based on the MaxEnt model and the ArcGIS spatial technology platform. This predictive data can provide research data for further studies on the distribution of tick populations in Inner Mongolia and the prevention and control of tick-borne diseases. Additionally, in-depth research on the current distribution status and trends of ticks in Inner Mongolia is of great significance for the monitoring and risk warning of tick-borne diseases in the region and the entire country.

## Methods

### Collection of tick geographical distribution data

A literature search was conducted in the CNKI (China National Knowledge Infrastructure), Wanfang, Baidu Scholar, PubMed, and Google Scholar databases using the keywords “Inner Mongolia” and “tick” for articles published between January 1, 1960, and November 30, 2022. By reading the titles, abstracts, and full texts of the articles, two reviewers filtered literature containing geographical distribution information of tick species in Inner Mongolia and extracted their geographical location information and distribution site coordinates. If the latitude and longitude were not indicated in the text, we determined the distribution site coordinates using the coordinate picker function on Google Maps based on the geographical location specified in the text. Based on a comprehensive review of existing literature reports, a summary of tick species distribution in Inner Mongolia has been compiled. The dominant tick species have been unequivocally determined through a thorough analysis of the number of distribution sites, ecological environments, records, and frequencies. To avoid overfitting due to overly concentrated distribution sites, ArcGIS 10.4 software was used to set a buffer zone with a radius of 10 km (consistent with the 10 km resolution of environmental climate data) for each acquired distribution point. Only one distribution site was retained in each 10 × 10 km grid.

### Selection of environmental factors

Environmental factors such as bioclimatic variables and geographical data were used in our study. We used the 19 bioclimatic variables from the WorldClim database [17]. The resolution of the 19 bioclimatic variable layers was 5 arc minutes (approximately 10 × 10 km pixels near the equator). To avoid overfitting multicollinearity, the 19 climate factors (Bio1–Bio19) were resampled using the sampling function in ArcGIS 10.4 software (Esri ArcMap; Esri, Redlands, CA, USA). The data were imported into SPSS 22.0 software (IBM Corporation, Armonk, NY), and Pearson's matrix was used for correlation analysis. Highly correlated variables were defined as those with a correlation coefficient |*r*|> 0.9 and were screened based on the contribution rate of environmental factors. Basic map data are derived from the Department of Natural Resources Standard Map Service System (https://www.webmap.cn/). Geographical data, including slope, aspect, and elevation data for China, were downloaded from the Geospatial Data Cloud (www.gscloud.cn), and slope and aspect variables were calculated using ArcGIS 10.4 software.

### Construction of the maximum entropy model

For the selected dominant tick species, the MaxEnt model was used to determine the key factors influencing tick distribution in Inner Mongolia. We randomly selected 75% of tick distribution sites as the training set and the remaining 25% as the test set to validate the model’s accuracy. The convergence domain limit was 10^−5^, the maximum number of iterations was 500, and 10 bootstrap calculations were performed for the current dominant tick species in Inner Mongolia using the MaxEnt model. The screened species distribution data and climate data were imported into the model. The jackknife method was selected in the environmental parameter settings to evaluate the weight of each ecological factor. We established univariate response curves for the distribution probability and environmental factors to determine the suitable range of environmental variable values.

Next, the accuracy of the model’s prediction results was validated using the receiver operating characteristic (ROC) curve in MaxEnt software. The area under the ROC curve (AUC value) was used to measure the model's predictive accuracy. Evaluation criteria were as follows: AUC values of 0.5–0.6 indicate failure, 0.6–0.7 indicate poor, 0.7–0.8 indicate fair, 0.8–0.9 indicate good, and 0.9–1.0 indicate excellent. The closer the AUC value was to 1, the more accurate the model's prediction results and the more significant the correlation between environmental factors and species distribution. The Jenks natural breaks classification method in ArcGIS 10.4 MaxEnt software was used to classify the habitat suitability levels of the four selected dominant tick species in Inner Mongolia.

### Construction of potential suitability areas model for dominant tick species

The future climate data were derived from the high-resolution climate model BCC-CSM2.MR in the Sixth Coupled Model Intercomparison Project (CMIP6) [18]. The BCC-CSM2-MR model included four components: atmosphere, land surface, ocean, and sea ice. Following the literature, the SSP245 scenario under the BCC-CSM2-MR model was selected [19]. Four future periods were used in the study: 2021–2040, 2041–2060, 2061–2080, and 2081–2100; SSP245 represents an upgrade of the RCP4.5 scenario based on SSP2, a medium forcing scenario. From the perspective of future greenhouse gas emissions and concentration trends, the RCP 4.5 scenario peaked in 2040 and stabilized by 2080. This greenhouse gas emission trend is consistent with China's future development trend and conforms to China's national conditions [20]. Therefore, we used the climate factors of the four periods under the SSP245 climate scenario as future environmental factors, along with the current geographical factors and distribution data for dominant tick species. These data were imported into MaxEnt software and run again to predict the habitat suitability of dominant tick species in Inner Mongolia under future environmental conditions.

### Changes in the centroid of suitable habitat of dominant species

In this study, we used the suitability areas of dominant tick species to investigate the spatial changes in the overall habitat suitability by examining the changes in their centroid from the near current and 2081–2100 time period. To obtain the centroid coordinates, we performed binary conversion on the distribution maps of the MaxEnt model’s habitat suitability using the ArcGIS 10.4 software. We revealed the changes in direction and distance of the suitable habitat for the dominant tick species by connecting the centroids of suitable habitat under various climate conditions.

## Results

### Tick species distribution in Inner Mongolia

**Fig. 1:**
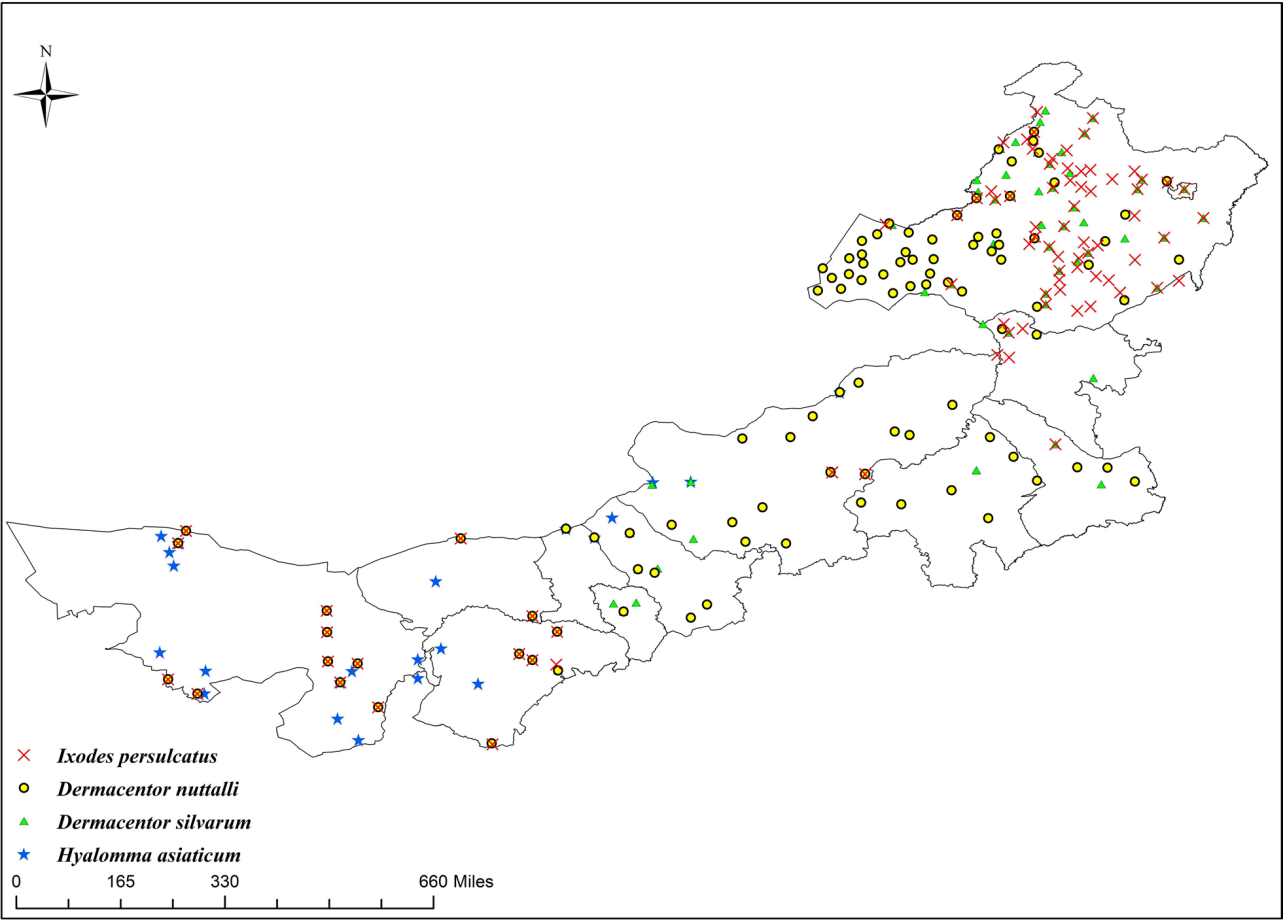
Distribution map of primary tick species in Inner Mongolia

In this study, a total of 5109 articles were retrieved, including 2483 Chinese articles (1740 from CNKI, 253 from Wanfang, 490 from Baidu Scholar) and 2626 English articles (1629 from PubMed, 997 from Google Scholar). After removing duplicates and articles with unclear distribution information that did not meet the criteria, 102 Chinese and 49 English articles were included (Additional file 1: Figure S1). The results showed that 23 tick species belonging to six genera were currently distributed in Inner Mongolia (Table 1). Considering the distribution of different tick species in various ecological environments and the available collection of distribution sites, we selected the four dominant tick species for subsequent modeling analysis. Among the four species, 39 articles and 172 distribution sites were included for *Dermacentor nuttalli*; 41 articles and 140 distribution sites for *Ixodes persulcatus*; 35 articles and 128 distribution sites for *Dermacentor silvarum*; and 23 articles and 38 distribution sites for *Hyalomma asiaticum*. We set a 10-km-radius buffer zone for the extracted tick distribution sites, and only one distribution site was retained in each buffer zone. Finally, 104 *D. nuttalli* distribution sites, 85 *I. persulcatus* distribution sites, 82 *D. silvarum* distribution sites, and 23 *H. asiaticum* distribution sites were determined (Fig. 1). The distribution sites of the two subspecies (*Hyalomma asiaticum asiaticum* and *Hyalomma asiaticum kozlovi*) were combined to establish the model.

**Table 1:** Distribution records of ticks in Inner Mongolia by references

Genus	Species	References
*Dermacentor*	*D. nuttalli*	[14, 15, 21–58]
	*D. silvarum*	[23, 26, 57, 59–92]
	*D. marginatus*	[22, 23, 93, 94]
	*D. niveus*	[28, 95]
	*D. sinicus*	[96]
*Hyalomma*	*H. asiaticum kozlovi*	[36, 38, 39, 45, 87, 88, 95, 97–102]
	*H. asiaticum asiaticum*	[16, 22, 23, 27, 41, 94, 103–106]
	*H. marginatum*	[54]
	*H. detritum*	[88, 107]
	*H. dromedarii*	[22, 23]
	*H. rufipes*	[108, 109]
*Haemaphysalis*	*H. concinna*	[26, 54, 58, 62–64, 67, 68, 74–79, 82, 84, 86, 91, 92, 110–115]
	*H. verticalis*	[45, 87, 95, 96, 116–119]
	*H. japonica*	[54, 84, 92]
	*H. longicornis*	[57, 93, 120, 121]
	*H. bispinosa*	[93]
*Rhipicephalus*	*R. turanicus*	[54, 94]
	*R. pumilio*	[88]
	*R. sanguineus*	[93]
*Ixodes*	*I. persulcatus*	[15, 29, 32, 37, 46, 48, 50, 55, 57, 58, 62–64, 67, 68, 75–77, 79, 81–84, 86, 89, 91, 92, 96, 110, 112, 118, 122–135]
	*I. crenulatus*	[121, 136]
*Argas*	*A. persicus*	[137–139]
	*A. japonicus*	[140]

### Key variables that influence tick distribution

To construct the MaxEnt model for tick distribution, 19 bioclimatic variables were screened, and six variables were selected for modeling analysis (Additional file 3: Table S1). We used the jackknife analysis to evaluate the impact of various environmental factors on the potential suitability areas for dominant ticks. The top five main variables and their contribution rates are shown in Table 2. The distribution of *D. nuttalli*, *I. persulcatus*, and *D. silvarum* is primarily influenced by temperature seasonality (Bio4), elevation (elev), and precipitation seasonality (Bio15), with contribution rates exceeding 10%. In contrast, the distribution of *H. asiaticum* was mainly affected by precipitation.

**Table 2:** Key variables contributing to tick distribution

	Tick species			
Key variables (contribution rate %)	*D. nuttalli*	*I. persulcatus*	*D. silvarum*	*H. asiaticum*
1st	Temperature seasonality = Bio4 (44.0)	Mean temperature of coldest quarter = Bio11 (29.9)	Temperature seasonality = Bio4 (43.2)	Annual precipitation = Bio12 (21.6)
2nd	Elevation = Elev (16.6)	Temperature seasonality = Bio4 (22.9)	Precipitation seasonality = Bio15 (14.6)	Elevation = Elev (18.1)
3rd	Precipitation seasonality = Bio15 (14.6)	Precipitation seasonality = Bio15 (16.0)	Elevation = Elev (11.4)	Precipitation of driest month = Bio14 (10.0)
4th	Slope = Slop (5.1)	Elevation = Elev (11.6)	Mean diurnal range = Bio2 (4.9)	Precipitation of warmest quarter = Bio18 (9.5)
5th	Precipitation of coldest quarter = Bio19 (4.2)	Mean temperature of driest quarter = Bio9 (3.9)	Mean temperature of coldest quarter = Bio11 (4.2)	Aspect = Aspe (9.0)

### Inferring potential suitability areas of ticks under near current and future conditions

The average AUC values of the ROC curve for the four tick species in 10 runs were 0.960, 0.964, 0.956, and 0.986, indicating that the model prediction accuracy is excellent in this study.

**Fig. 2:**
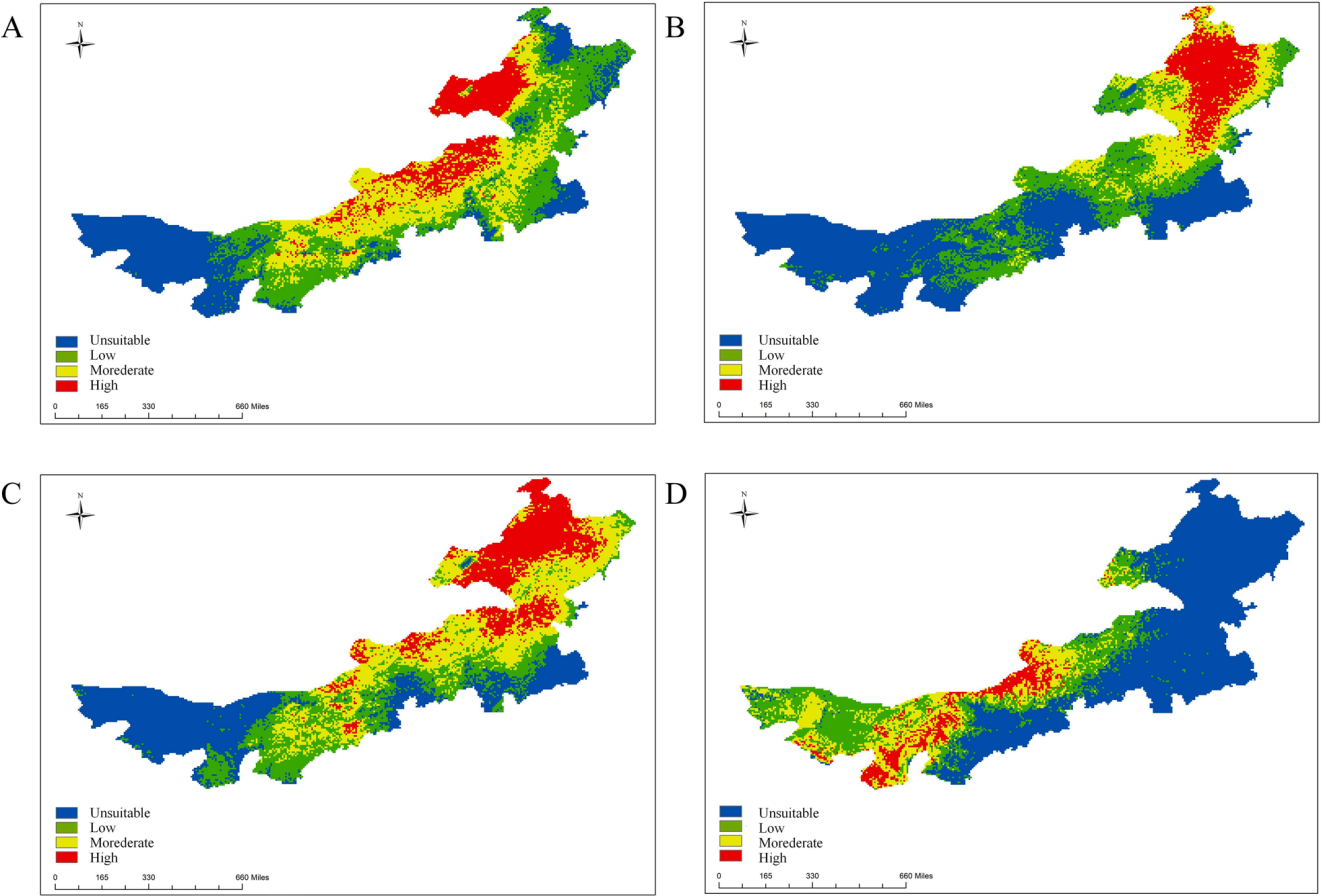
Predicted spatial distribution of tick suitability areas in Inner Mongolia. **A** *D. nuttalli*; **B** *I. persulcatus*; **C** *D. silvarum*; **D** *H. asiaticum*

**Fig. 3:**
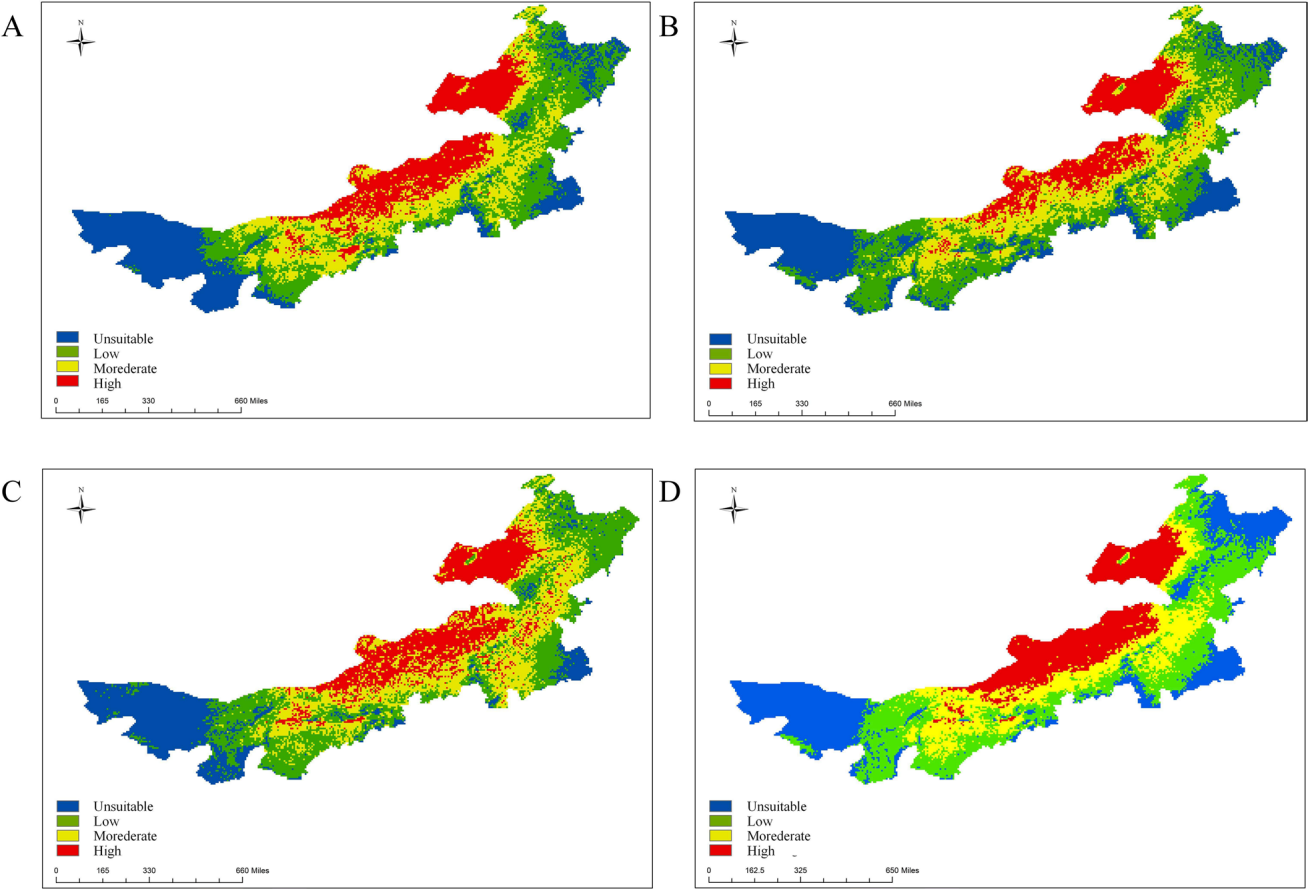
Predicted spatial distribution of *D. nuttalli* suitability areas in Inner Mongolia under future periods: **A** 2021–2040; **B** 2041–2060; **C** 2061–2080; **D** 2081–2100

MaxEnt software was used to obtain the potential suitability areas for the four dominant tick species in Inner Mongolia. The potential suitability areas were divided into four levels: high-suitability area, medium-suitability area, low-suitability area, and unsuitable area (Fig. 2). The results showed that the potential suitability areas for *D. nuttalli* were mainly distributed in Hulunbuir, Xilin Gol, and Ulanqab. In contrast, the unsuitable areas were located in Alxa, Tongliao, and Wuhai. Under future climate scenarios, the proportion of potential suitability and the high-suitability regions for *D. nuttalli* in Inner Mongolia increased (Fig. 3). Compared to the near current condition, the potential suitability area for *D. nuttalli* in Inner Mongolia increased by 168,900 km^2^, with new potential suitability areas distributed in Hulunbuir, Hinggan League, and Chifeng in 2081–2100. The potential suitability area decreased by 34,000 km^2^, and the loss of potential suitability areas mainly occurred in the northeastern part of Hulunbuir (Additional file 2: Figure S2A).

**Fig. 4:**
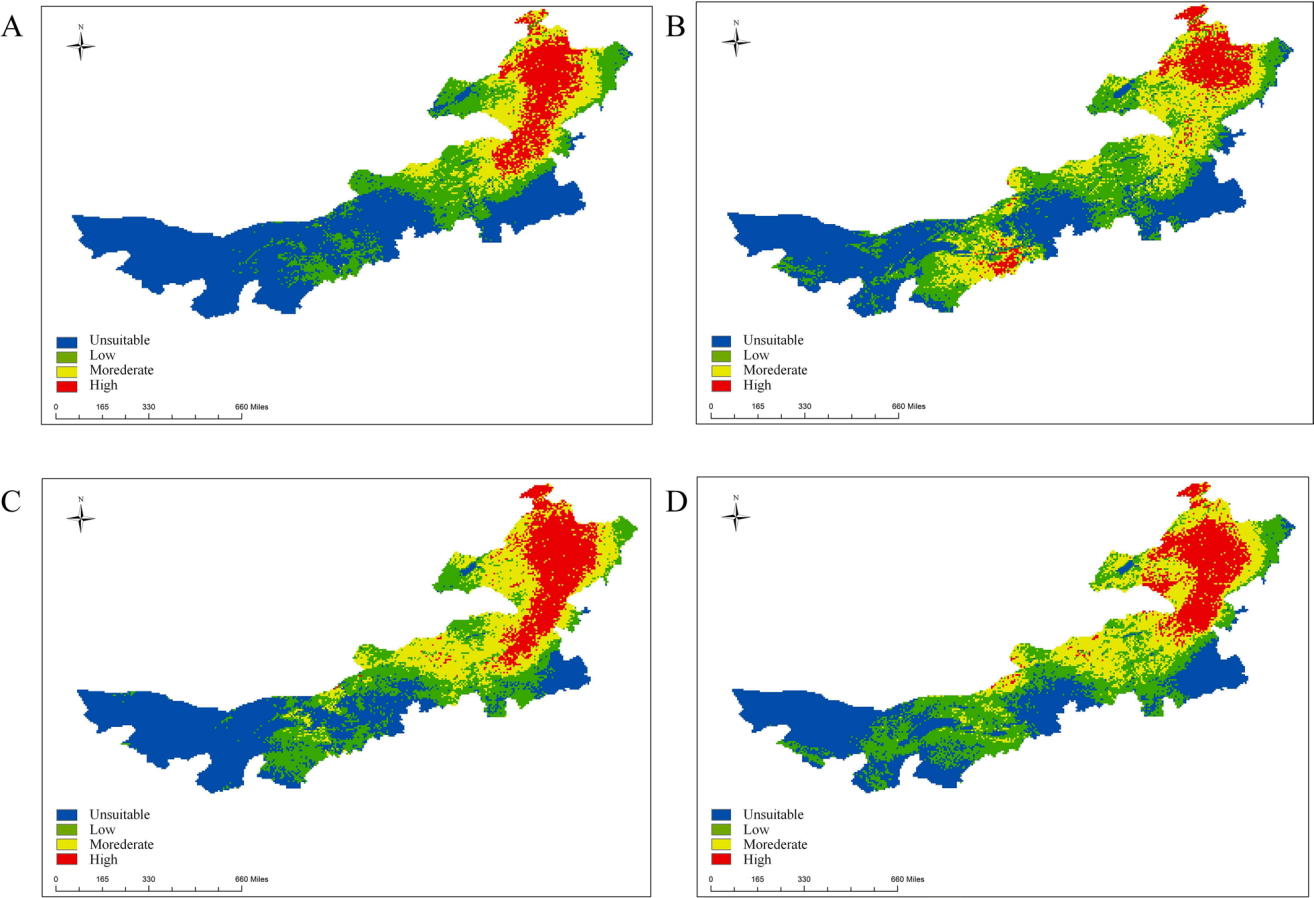
Predicted spatial distribution of *I. persulcatus* in Inner Mongolia under future periods: **A** 2021–2040; **B** 2041–2060; **C** 2061–2080; **D** 2081–2100

The potential suitability areas for *I. persulcatus* were mainly distributed in Hulunbuir, Hinggan League, and Xilin Gol, while the unsuitable areas were located in Alxa, Tongliao, and Ulanqab. Under future climate scenarios, the proportion of potential high-suitability areas for *I. persulcatus* in Inner Mongolia decreased and then increased (Fig. 4). Compared to the near current climate scenario, the potential suitability area for *I. persulcatus* in Inner Mongolia increased by 156,300 km^2^, with new potential suitability areas distributed in Chifeng, Xilin Gol, and Ordos in the 2081–2100 time period. The potential suitability area decreased by 34,000 km^2^, and the loss of potential suitability areas were mainly located in Ulanqab (Additional file 2: Figure S2B).

**Fig. 5:**
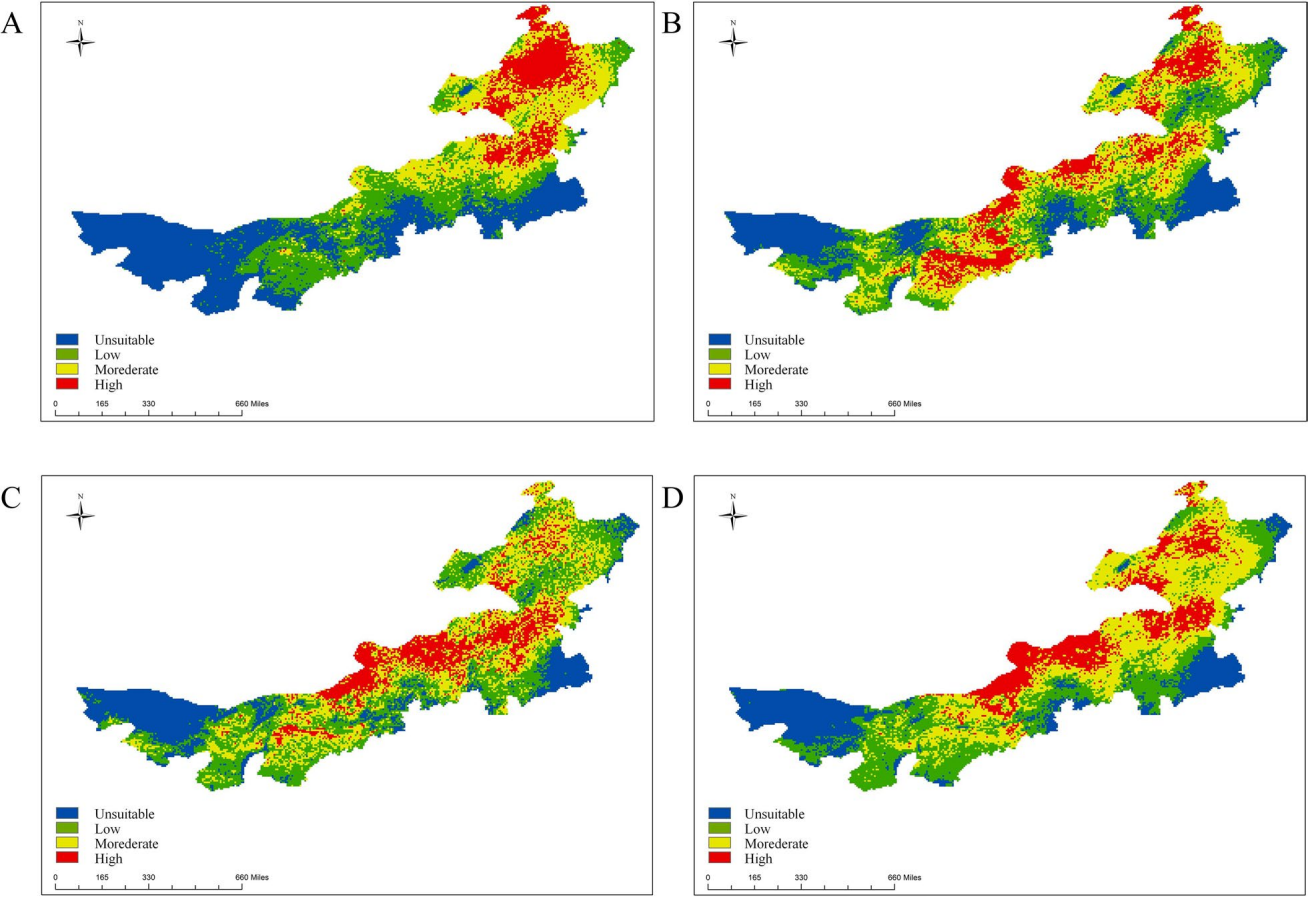
Predicted spatial distribution of *D. silvarum* in Inner Mongolia under future periods: **A** 2021–2040; **B** 2041–2060; **C** 2061–2080; **D** 2081–2100

The potential suitability areas for *D. silvarum* were found in Hulunbuir, Xilin Gol, and Baotou, while the unsuitable areas were mainly located in Tongliao and Alxa. Under future climate scenarios, the proportion of potential suitability areas for *D. silvarum* in Inner Mongolia increases in all time periods, except for 2021–2040 (Fig. 5). Compared to the near current, the potential suitability area for *D. silvarum* in Inner Mongolia increased by 135,300 km^2^, with new potential suitability areas distributed in Chifeng, Xilin Gol, and Bayan Nur in 2081–2100. The potential suitability area decreased by 24,800 km^2^, and the loss of potential suitability areas were mainly located in Hulunbuir and Tongliao (Additional file 2: Figure S2C) in the future climate scenarios.

**Fig. 6:**
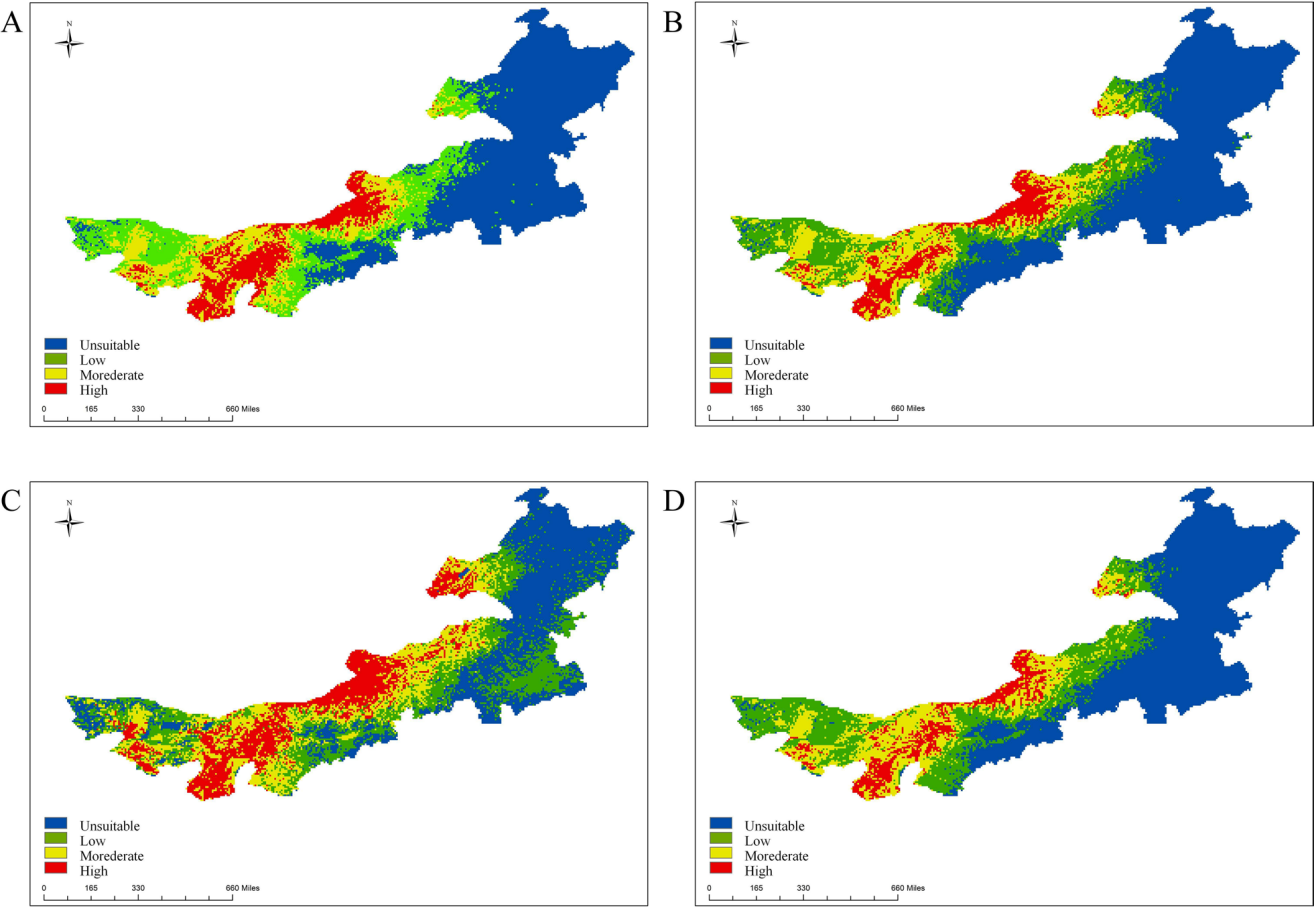
Predicted spatial distribution of *H. asiaticum* in Inner Mongolia under future periods: **A** 2021–2040; **B** 2041–2060; **C** 2061–2080; **D** 2081–2100

The potential suitability areas for *H. asiaticum* were mainly distributed in Alxa, Bayan Nur, and Baotou, while the unsuitable areas were primarily located in Hulunbuir, Chifeng, and Hinggan League. Under future climate scenarios, the proportion of potential high-suitability areas for *H. asiaticum* in Inner Mongolia increased (Fig. 6). Compared to the near current scenario, the potential suitability area for *H. asiaticum* in Inner Mongolia increased by 54,100 km^2^, with new potential suitability areas distributed in Ordos and Bayan Nur. The potential suitability area decreased by 9300 km^2^, and the loss of potential suitability areas were mainly located in Xilin Gol (Additional file 2: Figure S2D) in the future climate scenarios.

### Changes in the centroid of the potential suitability areas for dominant tick species in the near current and future period 2081–2100

**Fig. 7:**
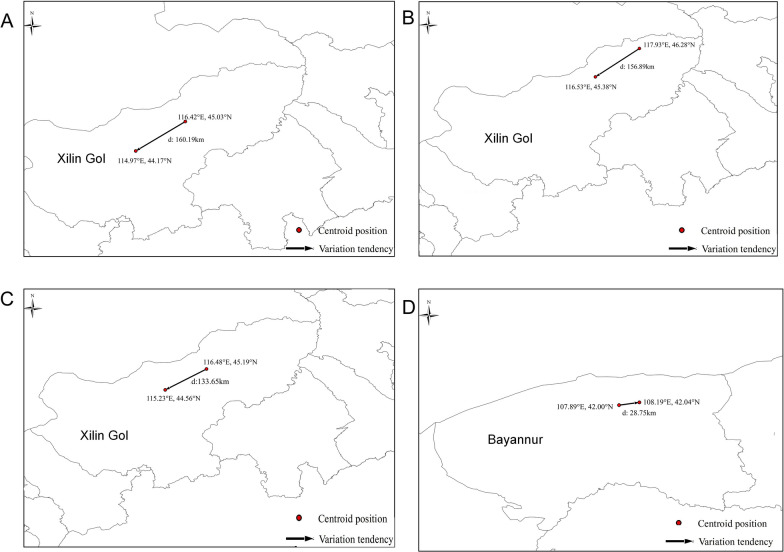
Changes in the centroid of the potential suitability areas for the dominant tick species in Inner Mongolia

The changes in the centroid of the potential suitability areas for dominant tick species in the near current and 2081–2100 time periods are shown in Fig. 7. By 2081–2100, the centroid of the potential suitability area for *D. nuttalli* shifted 156.89 km to the west-southwest direction, with a latitude change of approximately 0.9° and a longitude change of approximately 1.4°. There was also a westward expansion trend in the overall potential suitability area. By 2081–2100, the centroid of the potential suitability area for *I. persulcatus* shifted 156.89 km to the west-southwest direction, with a latitude change of approximately 0.9° and a longitude change of approximately 1.4°. The overall potential suitability area presented a westward expansion trend. By 2081–2100, the centroid of the potential suitability area for *D. silvarum* shifted about 133.65 km towards the west-southwest, with a latitude change of approximately 0.63° and a longitude shift of approximately 1.25°. The overall potential suitability area exhibited a westward expansion trend as well. By 2081–2100, the centroid of the potential suitability area for *H. asiaticum* moved about 28.75 km towards the east, with little change in latitude and a longitude change of approximately 0.3°. The overall potential suitability area demonstrated an eastward expansion trend.

## Discussion

The MaxEnt model for tick distribution was first reported in 2006 [10] and has since become widely used for predicting tick habitat. In this study, we used the latest version of climate factors and tick distribution data in Inner Mongolia to generate new insights into the potential environmental factors and spatial patterns for tick distribution in Inner Mongolia. Through a comprehensive literature review, we identified 23 species from six genera of ticks reported in Inner Mongolia over the past six decades. The most dominant tick species are *D. nuttalli*, *I. persulcatus*, *D. silvarum*, and *H. asiaticum*. Using the MaxEnt model, we predicted the potential suitability areas for these four dominant tick species across different ecological environments in Inner Mongolia.

The distribution results for different tick species indicate that species of the genus *Dermacentor*, including *D. nuttalli* and *D. silvarum*, are mainly distributed in arid grassland areas suitable for grazing cattle and sheep, such as Hulunbuir and Xilin Gol within Inner Mongolia. Hulunbuir is an important pastoral area in Inner Mongolia, known for its vast grasslands, making it a significant livestock production area and an essential habitat for *Dermacentor* and *Ixodes* genera. Several tick-borne pathogens have been reported in the Hulunbuir region, such as a high *Brucella* positivity rate of 4.00–87.80% in tick samples in 2020 [14] and a high tick infection rate of *Rickettsia* up to 85.14% in 2021 [141], posing a severe threat to human and animal health. Moreover, the grassland areas of Hulunbuir and Xilin Gol are home to a diverse and abundant population of rodents and grazing animals, which can support a high density of ticks by serving as natural hosts. Moreover, local herders typically adopt grazing prohibition, rest, and rotation systems [142], which results in large-scale mobility of local wildlife and livestock. The migration of animal populations can lead to the passive movement of ticks that feed on them, thus expanding the potential suitability areas and distribution ranges of these tick species.

In contrast, *I. persulcatus* is mainly distributed in the Greater Khingan Range in northeastern China, adjacent to Russia. This region is characterized by high vegetation coverage and is one of China's important forestry and animal husbandry bases [54]. The forest–grassland transition zone of the Greater Khingan Range is a typical large-scale community-interlaced area in northern China and a key area where the Greater Khingan Range forest borders the Hulunbuir grassland. The transition zone has high biodiversity, frequent material flow, and strong spatio-temporal dynamic changes, which facilitates the development and reproduction of ticks [143]. *Haemaphysalis asiaticum* is mainly distributed in arid desert and desert environments in Inner Mongolia, such as Alxa and Bayan Nur. Alxa is located in the western part of the Inner Mongolia Autonomous Region and has a temperate continental climate. The geographical area in question exhibits high temperatures, a limited amount of rainfall, and arid conditions. This environment promotes significant evaporation, and noteworthy fluctuations in temperature are common throughout the day. Additionally, sand and dust storms frequently occur, providing favorable conditions for the development and reproduction of *H. asiaticum* [144]. In addition, *H. asiaticum* parasitizes multiple hosts, with larvae feeding on mice, rabbits, hedgehogs, and other small animals typically found in arid areas [145]. In contrast, nymphs and adults usually parasitize large animals such as camels, cattle, horses, sheep, and wild boars [146]. Therefore, host distribution is also a crucial factor for the survival of *H. asiaticum* in arid environments.

Based on the analysis of the primary climatic and environmental variables, it is evident that temperature seasonality standard deviation (Bio4), elevation (elev), and precipitation seasonality (Bio15) significantly influence the distribution of *D. nuttalli*, *I. persulcatus*, and *D. silvarum*. Meanwhile, we also found considerable overlap in their suitable habitat ranges among these species. The probability of these tick species' distribution reaches its maximum when the temperature seasonality standard deviation (Bio4) is 1719.62, 1939.03, and 1891.02, respectively. It can be inferred that these species are more sensitive to temperature. This finding is consistent with the results of Yang et al., which indicated that the probability of *D. nuttalli* distribution is more susceptible to temperature than other tick species (*Ixodes granulatus*, *Haemaphysalis longicornis*, and *Dermacentor marginatus*) [147]. The probabilities of the three tick species’ distribution are within the high-suitability range when elevations are 530.84–848.75 m, 581.65–907.59 m, and 493.56–961.61 m, respectively. This demonstrates that *D. silvarum* has a lower elevation requirement for subsistence and reproduction, which is consistent with our model proposing that the high-suitability area for *D. silvarum* is larger than that for the other three tick species. In the near current scenario, the high-suitability area for *D. silvarum* accounts for 19.13% of the total area of Inner Mongolia.

Notably, when Bio15 (precipitation seasonality) is approximately 118 mm, the suitability for three tick species is the highest. This could be a key reason for the overlapping distribution of these tick species in arid grassland regions. Wang et al. revealed the significant impact of Bio15 on *D. nuttalli* distribution [148]. In contrast to the three ticks mentioned earlier, *H. asiaticum* is primarily affected by precipitation-related climatic factors. A study on *H. asiaticum* distribution in Xinjiang showed that a longer summer and shorter winter is an ideal habitat for the species. A lower precipitation variability level corresponds to higher suitability, as stable precipitation helps maintain relatively stable air humidity [149]. Under these conditions, *H. asiaticum* can absorb water vapor from the air during their host-seeking period, maintaining hydrological balance for subsistence.

Considering the overall increase and decrease in the potential suitability areas inferred for the four tick species, their suitability areas are expected to expand in the near current and the future period of 2081–2100. There are several studies that are consistent with our model. For instance, Yang et al. revealed that the northeastern forest region would become warmer and more suitable for *D. nuttalli* due to global warming and land-use changes [147]. Additionally, Ma et al. indicated that suitable areas for *I. persulcatus* will increase in Inner Mongolia by 2070 [150]. Thus, it is evident that the combined effects of climate change, human activities, land use, and vector population growth will lead to the expansion of suitable habitat areas for the dominant tick species in Inner Mongolia. During 2081–2100, the centroid of suitable habitats for *D. nuttalli*, *I. persulcatus*, and *D. silvarum* is expected to shift westward, with local expansion in parts of Alxa, Bayannur, and Ordos. The centroid of suitable habitat areas for *H. asiaticum* will migrate towards the east-northeast, with newly suitable habitat areas emerging in parts of Ordos and Bayannur. This shift may be related to the reforestation, forest protection, and afforestation projects undertaken in desert areas such as Alxa, as well as the establishment of forest ecosystem benefit compensation systems and afforestation subsidy pilot projects, which have reduced the unused land area in sandy, Gobi, and desert regions [151].

The present study has utilized a comprehensive dataset gathered from various literature sources spanning a broad temporal and geographical range. The majority of reports included in the study were based on either morphological or molecular identification of tick species, and the MaxEnt distribution modeling was performed based on these data. Although there is the possibility of prediction distribution biases due to repeated sampling. By removing duplicate records at the same location and ensuring a minimum distance between sampling records, this study has effectively corrected the geographical sampling bias in the tick distribution dataset. However, it is important to note that the model only provides potential areas where a given species may survive, which may not necessarily represent actual distributions or species abundance. Our model considers only abiotic factors and does not consider the influence of hosts and other biotic factors. For example, the analysis does not include influential factors such as the distribution of animal hosts and human social activities, which may result in tick distribution found in unsuitable habitat areas. Future investigations could therefore incorporate tick-host analysis and further combine multiple modeling and evaluation methods to improve the limitations of the current MaxEnt model.

## Conclusion

In summary, the present study has unveiled the extant distribution of tick species in Inner Mongolia through analysis of available data sources. Moreover, utilizing the MaxEnt and ArcGIS spatial technology platforms and taking into account the tick distribution site data and pertinent bioclimatic variable data, we have projected the distribution of the four dominant tick species under the near current and future periods. These findings are important for tick research and monitoring the spread of tick-borne diseases in the region.

### Supplementary Information

**Additional file 1: Figure S1** Flow diagram of literature search and inclusion.

**Additional file 2: Figure S2** Changes in the potential suitability areas for the four dominant tick species under the near current and 2081–2100.

**Additional file 3: Table S1** Environmental and bioclimatic variables for the four dominant tick species distribution models by MaxEnt.

## Data Availability

Not applicable.
