# Supplementary figures and images for: The current distribution of tick species in Inner Mongolia and inferring potential suitability areas for dominant tick species based on the MaxEnt model
Source: Parasit Vectors. 2023 Aug 16;16:286. doi: 10.1186/s13071-023-05870-6 (PMC10428659; doi:10.1186/s13071-023-05870-6)

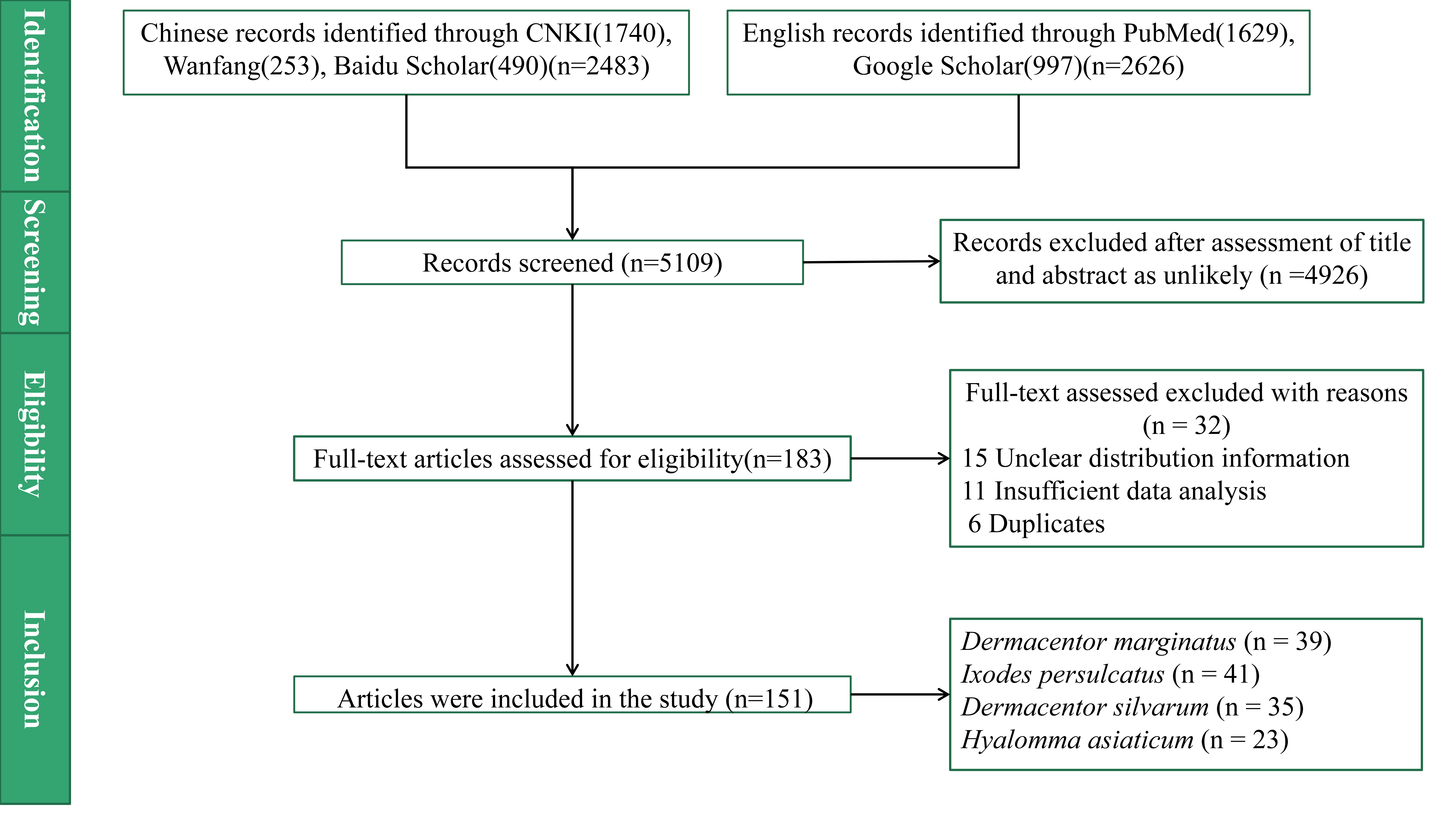

Supplement: Supplementary file 1 — Additional file 1: Figure S1 Flow diagram of literature search and inclusion. [file 13071_2023_5870_MOESM1_ESM.jpg]

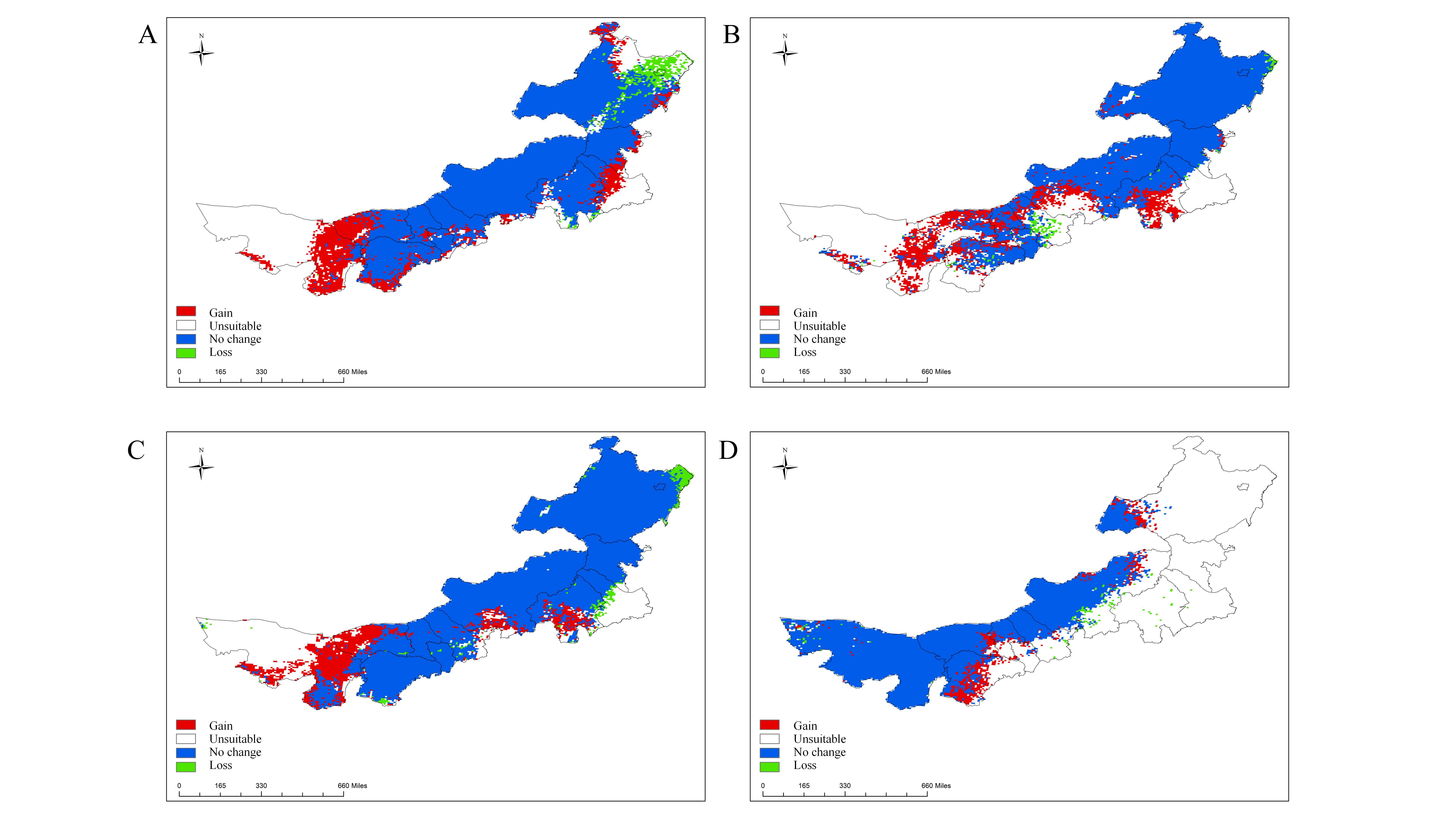

Supplement: Supplementary file 2 — Additional file 2: Figure S2 Changes in the potential suitability areas for the four dominant tick species under the near current and 2081–2100. [file 13071_2023_5870_MOESM2_ESM.jpg]
